# Interacting effects of phytohormones and fruit pruning on the morpho-physiological and biochemical attributes of bell pepper

**DOI:** 10.1038/s41598-024-65855-y

**Published:** 2024-06-26

**Authors:** Sayedeh Fatemeh Moosavi, Maryam Haghighi, Iman Mirmazloum

**Affiliations:** 1 Department of Horticulture, College of Agriculture, Isfahan University of Technology, Isfahan, Iran; 2 Department of Plant Physiology and Plant Ecology, Institute of Agronomy, Hungarian University of Agriculture and Life Sciences, Ménesi Str. 44, 1118 Budapest, Hungary

**Keywords:** Capsicum, Antioxidant capacity, Yield, Growth regulators, Fruit quality, Vitamin C, Auxin, Gibberellins, Photosynthesis, Plant physiology, Plant reproduction

## Abstract

Several factors, such as pruning and phytohormones, have demonstrated an influence on both the quantity and quality in the bell pepper. A factorial experiment using a completely randomized design was conducted on the Lumos yellow bell in a greenhouse. Treatments were the fruit pruning (0, 10, and 30%) and foliar application of phytohormones auxin (AUX) and gibberellic acid (GA_3_) at concentrations of 10 µM AUX, 10 µM GA_3_, 10 µM AUX + 10 µM GA_3_+, and 20 µM AUX + 10 µM GA_3_ along with controls. The plants were sprayed with phytohormones in four growth stages (1: flowering stage when 50% of the flowers were on the plant, 2: fruiting stage when 50% of the fruits were the size of peas, 3: fruit growth stage when 50% of the fruits had reached 50% of their growth, and 4: ripening stage when 50% of the fruits were at color break). The results of the present investigation showed that pruning rate of 30% yielded the highest flesh thickness and vitamin C content, decreased seed count and hastened fruit ripening. The use of GA_3_ along with AUX has been observed to augment diverse fruit quality characteristics. According to the results, the application of 10% pruning in combination with 20 µM AUX and 10 µM GA_3_ demonstrated the most significant levels of carotenoids, chlorophyll, and fruit length. The experimental group subjected to the combined treatment of 30% pruning and 10 µM AUX + 10 µM GA_3_ showed the most noteworthy levels of vitamin C, fruit weight, and fruit thickness. The groups that received the 10 µM GA_3_ and 20 µM AUX + 10 µM GA_3_ treatments exhibited the most favorable fruit flavor. According to the research results, the implementation of hormonal treatments 10 µM AUX and 10 µM AUX + 10 µM GA_3_ in combination with a 30% pruning strategy resulted in the most advantageous yield of bell peppers.

## Introduction

The cultivation of sweet pepper (*Capsicum annuum*) holds significant importance as a fruit crop in regions characterized by tropical and subtropical climates and in greenhouse cultivation systems worldwide. Sweet pepper fruit is known for its valuable phytochemicals, such as antioxidants, vitamins, carotenoids, flavonoids, and phenolic acids^1^. The antioxidant capacity of fruits differs because of various external factors such as light intensity, temperature, and drought, as well as internal factors such as fruit cultivar (genotypes)^2^. Plants exhibit coordinated behaviors by synthesizing and utilizing different growth regulators. Phytohormones are a class of organic and naturally occurring compounds that exert physiological effects at low concentrations, regulating plant growth and performance by either inhibiting or stimulating various physiological processes, from the regulation of stomata aperture to the development of fruits^3^.

The auxins (AUX) have been found to be involved in various growth regulatory aspects such as cell division, elongation of cellular dimensions, fruit development, promotion of flowering, and partitioning of assimilates, which are the products of photosynthesis^4^. Several research studies have demonstrated that phytohormones, specifically auxins, have significant effects on the uptake and movement of nutrients within plants by controlling the sink function of developing tissues^5^. Plants that are exposed to IAA have higher pigment content, photosynthetic rate, stomatal conductance, and accumulation of sugars including glucose, fructose, and total soluble sugars^6–8^. Furthermore, the rise of photosynthesis is facilitated by auxin through the expansion of leaf vein density, which is achieved through carefully arranged placement, thereby leading to an enhancement in the photosynthetic capacity of the leaf^9^. IAA treatment also increases nitrate reductase activity and encourages development, as observed in *Solanum melongena*^10^. The concentration of mineral nutrients in plant roots and leaves is increased by IAA as well as by its precursors, such as L-tryptophan and indole^11,12^. Additionally, auxin increases both enzymatic (such as catalase, ascorbate peroxidase, and superoxide dismutase) and non-enzymatic (such as glutathione and ascorbate) antioxidants, and it decreases ROS levels and lipid peroxidation^13,14^.

Gibberellic acid (GA_3_) is another important growth regulator with an indispensable role in plant development. According to Tiwari et al.^4^, the application of gibberellin has been found to facilitate the formation of pepper fruits by promoting fruit thickening and enhancing the development of super fruit, with desirable quality suitable for higher level marketing^15^. Exogenous GA_3_ application ameliorated fruit size, followed by a subsequent increase in yield and enhancement of fruit quality^16^. The use of absorbed nutrients to enhance photosynthetic efficiency, a decreased level of respiration, and an improved transfer and metabolism of metabolites are all claimed to be facilitated by gibberellin^15^. Foliar application of GA_3_ resulted in increased levels of TSS and total sugar in Aonla fruits^17^. According to Singh and Singh^18^ the application of GA_3_ at 150 ppm resulted in a higher vitamin C content in bell pepper at harvest time.

Normal fruit growth is influenced by the coordinated activity of AUX and gibberellin^4^. The availability of both phytohormones is imperative and crucial for the typical development of fruits^19^. It has been claimed that the auxin acts as a primary signal a principal element for fruit set induction, which requires downstream gibberellin biosynthesis to reduce the abscission^4^. According to Saha^20^, the concurrent use of NAA (25 ppm) and GA_3_ (40 ppm) yielded superior results in terms of TSS and vitamin C content when compared to their individual application. In another study, the application of GA_3_ and NAA at 10 and 20-ppm concentrations, respectively, resulted in the highest total soluble solid content in *Solanum melongena* L.^21^.

Pruning, which involves the complete or partial removal of branches, roots, bark, leaves, flowers, and fruits, is a known technique for influencing and controlling plant growth and yield. Pruning confers several advantages, such as enabling the penetration of light and air into the branches, regenerating the plant organs, reducing excessive vegetative growth, stimulating reproductive growth and fruit production, and promoting equilibrium between the root, stem, and areal parts of the plants. Pruning excess branches from plant facilitates elongated growth and enhances the quality of fruits in proximity to the primary stem^22^. Pruning also serves the purpose of achieving a balance between the expected yield and overall vigor of the plants^23,24^. According to Alsadon^23^, reducing the number of branches resulted in an improvement in the quality of bell pepper manifested in both the average weight and overall quality characteristics of the fruits.

The present study aimed to investigate the combined effects of different fruit pruning levels and the foliar application of different phytohormones (AUX and GA_3_) alone or in combination on the morphological and qualitative characteristics of Lumos yellow bell pepper.

## Materials and methods

### Experimental design

Based on our preparatory and initial trials with a wider range of treatments (pruning at 10, 20, and 30%; and eight different combinations of hormonal application), the best treatments were chosen, to conduct a factorial experiment in the form of a completely randomized design (CRD) in a plastic greenhouse with bell pepper *Capsicum annum* cv. yellow Lumos. The treatments of this experiment included pruning at three levels: non-pruning (control), 10% pruning, and 30% pruning, and four different combinations of phytohormones including control, 10 µM auxin (AUX1), 10 µM GA (GA_3_), 10 µM auxin + 10 µM GA (AUX1 + GA_3_), and 20 µM auxin + 10 µM GA (AUX2 + GA_3_) in triplicates. Morphological and biochemical parameters were measured and analyzed.

The seeds of the bell pepper variety were obtained from Syngenta Company and sown in peat moss and perlite seed trays. Seedlings with four to six true leaves were transferred to the greenhouse and planted at a density of three plants per square meter. Plants were grown in nutrient-rich soil under environmental temperatures of 26 ± 3°C (day) to 18 ± 3°C (night) in December. Irrigation was carried out in strips every other day and the relative air humidity was recorded to be at 50–55%.

Foliar spraying of phytohormones was applied at four phenological stages including the flowering stage, fruit set stage, fruit development stage (when 50% of the fruits had reached 50% of their expected growth), and ripening stage (when 50% of the fruits were at color break). Plants were sprayed to the point of runoff (until every leaf was wetted up to the dripping point).

Plants were irrigated in 2 days intervals sequentially with tap water and a fertigation solution (5mM KNO_3_, 2 mM KH_2_PO_4_, 1.5 mM CaSO_4_∙2H_2_O) in 80:20 ration. The foliar spraying of microelements (1 mM MgSO_4_∙6H_2_O, 20 µM Ferric sodium EDTA, 25 µM H_3_BO_3_, 2 µM MnSo_4_∙7H_2_O, 2 µM ZnSO_4_∙7H_2_O, 0.5 µM CuSO_4_∙5H_2_O, 0.1 µM NiSO_4_∙6H_2_O, 0.25 µM Na_2_MoO_4_∙2H_2_O; EC: 1.25 µS/cm) was performed^25^ every three weeks. The harvest was gradually started 80–90 days plantation in the greenhouse.

To perform the fruit pruning operation, the total number of fruits per plant was counted and averaged, and then 10% and 30% pruning was performed on each plant in a uniform manner. As a common practice, the old leaves at the bottom of the plant were also regularly cut to facilitate air and light circulation.

### Measurements and observations

#### Fruit yield and morphology (length/diameter/thickness/firmness)

The fruit samples were subjected to a quantitative analysis, where their numerical count and weight were determined. In addition, the physical features of the fruit, including the diameter (the distance across the fruit shoulders), length (the maximum length without the peduncle), and pericarp thickness were measured. Fruit shape (length/diameter), total seed weight per fruit, and firmness were recorded at the time of harvest. The seed weight (g) was determined by removing the intact receptacle from which the seeds were separated and subsequently weighed. Fruit yield was determined by the weight of the harvested fruits per plant.

Three different equatorial zones of the ripe fruits of each treatment were randomly selected for the measurement of pericarp firmness using a Penetrometer (model OSK-I-10,576, Ogawa Seiki Co. Ltd., Tokyo, Japan) at harvest time, as described by Pathaveerat et al.^26^. The force generated against the penetration probe was recorded and used for data analysis and the results were expressed in Newton (N).

#### Color index

A digital camera (Cannon EOS 6D) was used to take photos of the fruits in a constant position and equal lighting conditions. Subsequently, image processing and coding techniques were employed through the use of the Photoshop CS5 program to extract color components and other relevant information from the images. Finally, the L*, a*, and b*, indices were recorded as pixels^27^.

#### Color break and ripening time

The date on which the color halos appeared on fruits was recorded for each plants under different treatments and designated by ribbons. The ripening date was also recorded for each fruit and the time interval the color formation and ripening was determined.

#### Fruit relative water content (RWC)

RWC was determined following the methods described by Lopez-Serrano et al.^28^. After the fresh weight (FW) measurement, fruit disks were subjected to float in distilled water for 12 h. The turgid weight (TW) of the fruits was determined before drying them at 80 °C for 48 h.

#### Total carbohydrates content of fruits

Total soluble carbohydrates were measured using the anthrone method. Fruit tissue (1.0 g) was homogenized in 5 ml of 95% ethanol, then centrifuged at 3500 rpm for 10 min at 10°C, the supernatant was collected in a new tube. The extraction was repeated three times when 70% ethanol was used in the second and third round and the supernatants were combined in one test tube. Finally, the obtained extract (100 μl) was added to 3 ml of anthrone solution (0.15 g of anthrone + 100 ml of 70% sulfuric acid) and the mixture was heated at 95°C for 15 min. After cooling, the absorbance at 625 nm was recorded using a spectrophotometer (UV 160A- Shimadzu Corp., Kyoto, Japan). Glucose was used as a standard to quantify total soluble carbohydrates^29,30^.

#### Total carotenoid and chlorophyll content in fruits

Fruit disk samples (6–8 mm in diameter) were homogenized in 80% acetone in a mortar with a pestle. Small amounts of magnesium carbonate (MgCO_3_) was added to reach the alkaline condition. The extract was centrifuged at room temperature for 5 min at 12,000 rpm. The optical density of the supernatant was measured at 661.6, 644.8, and 470 nm using a spectrophotometer (UV 160A-Shimadzu Corp., Kyoto, Japan) in cuvettes^31^. The following equations were used to determine the chlorophylls content in mg per 100 g fresh weight of fruit.

#### $$\begin{gathered} {\text{Chlorophyll }}a \, = \, \left( {{19}.{3 } \times {\text{ A663 }} - \, 0.{86 } \times {\text{ A647}}} \right){\text{ Volume }}/{ 1}00{\text{ g}} \hfill \\ {\text{Chlorophyll }}b \, = \, \left( {{19}.{3 } \times {\text{ A647 }} - { 3}.{6 } \times {\text{ A663}}} \right){\text{ Volume }}/{ 1}00{\text{ g}} \hfill \\ {\text{Carotenoids }}\left( {\mu {\text{g 1}}00{\text{ g}}^{{ - {1}}} {\text{FW}}} \right):{ 1}00 \, \left( {{\text{A47}}0} \right) \, {-}{ 3}.{27 }\left( {{\text{mg g}}^{{ - {1}}} {\text{Chl}}. \, a} \right) \, {-}{ 1}0{4 }\left( {{\text{mg g}}^{{ - {1}}} {\text{Chl}}. \, b} \right) \, /{ 227} \hfill \\ \end{gathered}$$2.2.7. Fruit vitamin C content

The Ascorbic acid content of the bell pepper fruits was determined according to a method described by Njoku et al.^32^ with minor changes. Fresh fruit samples (1 g) were homogenized with 20 ml of 0.4% oxalic acid in a flask. After incubation for 5 min at room temperature and filtration, 100 μl of the filtrate was weighed into a microtiter plate. Then, 200 μl of 0.8 mg/ml 2,6-dichlorophenolindophenol was added, incubated for 5 min, and the absorbance at 520 nm was measured with a spectrophotometer (UV 160A—Shimadzu Corporation, Kyoto, Japan). To determine the ascorbic acid content of fruit samples, standard solutions of ascorbic acid with different concentrations (0.2, 0.4, 0.6, 0.8, and 1.0 mg/ml) were prepared. Results were expressed in mg ascorbic acid per 100 g of fruit sample using the calibration curve (R^2^ = 0.935) reported by Idris et al.^33^.

#### Antioxidant capacity

The determination of the antioxidant activity of pepper fruits was done by the methodology outlined by Koleva et al.^34^. Following the addition of 200 μl of an analytical sample solution and 800 μl of a Tris–HCl buffer (pH 7.4) 1 ml of DPPH solution was added to the test tube and mixed for 10 s. The solution was then stored at room temperature in a light-free environment. After exactly 30 min, the absorbance of the solution was measured at 517 nm using a spectrophotometer (UV 160A-Shimadzu Corp., Kyoto, Japan). The blank consisted of 1.2 ml of ethanol and 800 μl of Tris–HCl buffer. The absorbance after adding the analytical sample was defined as As, and the absorbance after adding ethanol instead of the sample was defined as Ac. The inhibition rate (%) was calculated using the formula:

$${\text{Inhibition rate }}\left( \% \right) \, = \, \left( {\left( {{\text{Ac}} - {\text{As}}} \right)/{\text{Ac}}} \right) \times {1}00.$$

#### Total soluble solids (TSS):

Solids (TSS) in pepper fruit were measured by using a digital refractometer (k-0032, Japan). A drop of the juice was placed on the lens and the reading was taken in degrees Brix (^◦^Bx) and expressed as % soluble solids content in the fruit. Calibration made with distilled water and the lens was carefully rinsed, between samples, with distilled water twice^35^

#### Fruit titratable acidity (TA)

The TA, which was expressed in percentage acetic acid, was determined by an automatic potentiometric titrator (907 GPD titrino, Metrohm, Switzerland) with 0.05 M NaOH until the pH value was reached 8.1^36^. The TA of acidified bell peppers was calculated by Equation bellow.

$$TA \left(as \% acetic acid\right)=\frac{C\times V\times K}{m}\times 100$$

where C is the concentration of NaOH (0.1 M); m is the weight of acidified chili peppers; V (ml) is the volume of used NaOH; and K is the conversion factor of citric acid.

#### Fruit flavor (TSS/TA)

For fruit flavor estimation we used the TSS/TA formula. TSS is for total soluble solids, and TA stands for organic acids in percentage in 100 ml of extract, and this association has a positive correlation with the fruit's edibility^37^.

### Statistical analysis

The research was conducted under a factorial experiment using a complete randomized design (CRD) with three replications. The data were analyzed using Statistix 8 software (Tallahassee, FL, USA). The data received a two-way analysis of variance (ANOVA), after the homogeneity assumptions were confirmed by Levene’s test. The mean values were assessed using the least significant difference (LSD) test at *P* < 0.05. Principal component analysis (PCA) plot was created using Statgraphics Centurion, Version XVI to visualize the relationship between the measured variables and the samples using a bi-plot.

## Results

### Correlation analysis of variances amongst the studied traits

According to the statistical analysis of the data on morphophysiological traits, pruning treatment had a significant effect on the firmness of the stem end (*p* < 0.01), and on other morphophysiological traits (excluding fruit weight and diameter) based on the LSD test (*p* < 0.05). Hormone treatment had a strong significant effect on firmness of the stem end and firmness of the middle fruit (*p* < 0.01), and on weight, length, fruit thickness, yield, and color index b (*p* < 0.05). However, the hormone treatments did not significantly affect the other morphophysiological characteristics. The interacting effect of pruning and hormones was significant (*p* < 0.05) for all morphophysiological traits (Table 1).

**Table 1:** Analysis of variance of phytohormones and pruning effects on growth traits and morphophysiological characteristics of bell peppers.

Source	df	Fruit weight	Fruit length	Fruit diameter	Fruit thickness	Seed weight	Yield	Firmness of stem end	Firmness of fruit	Firmness blossom end	a*	b*	L*
Pruning	2	1243.9 ^n.s^	1.38*	0.33 ^n.s^	9.02*	26.99*	190,434*	1.26**	3.22*	0.83*	68.86*	116.86*	201.62*
Hormone	4	3335.5*	0.7 ^n.s^	1.08*	6.33*	69.69*	27,984*	0.95**	0.98**	0.186 ^n.s^	10.31 ^n.s^	18.75*	18.97 ^n.s^
Pruning × hormone	8	857.11*	0.50*	0.26*	3.49*	28.86*	56,384*	0.32*	0.71*	0.483*	18.22*	26.33*	41.59*
Total	44	55,274.20	28.35	15.91	124	851.99	39	14.38	23.19	11.79	766.80	842.8	1375.91
Cv		12.59	8.01	6.52	14.55	24.75	55.22	15.65	16.96	15.68	16.91	4.78	6.45

*ns* not significant, *df* degree of freedom.

*Significant at *p* < 0.05 and **significant at *p* < 0.01 probability level.

Based on the analysis of variances, pruning had a strong and positively significant effect on the soluble solids of the fruits (*p* < 0.01). Fruit vitamin C content and fruit flavor were significantly correlated with the applied treatment (*p* < 0.05). The phytohormone applications did not have a significant effect on fruit ripening time, carbohydrate and carotenoid levels, but it showed significant effect (*p* < 0.05) on other physiological characteristics investigated in this research (Table 2). The mutual effect of pruning and hormones had a significant effect on all physiological traits studied in this study except carbohydrates.

**Table 2:** Analysis of variance of phytohormones and pruning effects on some physiological characteristics of bell peppers.

Source	df	Color break	Ripening time	RWC	Total carbohydrate	Chlorophyll *a*	Chlorophyll *b*	Carotenoid	Vitamin C	Antioxidant capacity	TSS	TA	TSS/TA
Pruning	2	33.08 ^n.s^	22.46 ^n.s^	80.93*	18.09 ^n.s^	2.53 ^n.s^	1.20 ^n.s^	0.21 ^n.s^	685.58*	3.01 ^n.s^	9.07**	6.34 ^n.s^	25,613*
Hormone	4	63.74*	41.18 ^n.s^	68.06*	41.64 ^n.s^	8.09*	3.95*	0.17 ^n.s^	447.88*	2.72*	3.47*	1.35*	28,545*
Pruning × Hormone	8	24.06*	28.68*	42.95*	32.99 ^n.s^	1.76*	5.62*	0.43*	320.20*	4.05*	2.05*	1.06*	20,166*
Total	44	1784.98	1577.20	1638.01	61.97	7.17	1.49	0.18	143.45	2.22	89.42	6.43	10,702
Cv		12.66	10.62	6.08	17.90	16.02	114.43	54.20	13.74	1.63	17	27.67	37.81

*ns* not significant, *df* degree of freedom.

*Significant at *p* < 0.05 and **significant at *p* < 0.01 probability level.

### Interacting effects of pruning and phytohormones on fruit quality characteristics

**Figure 1:**
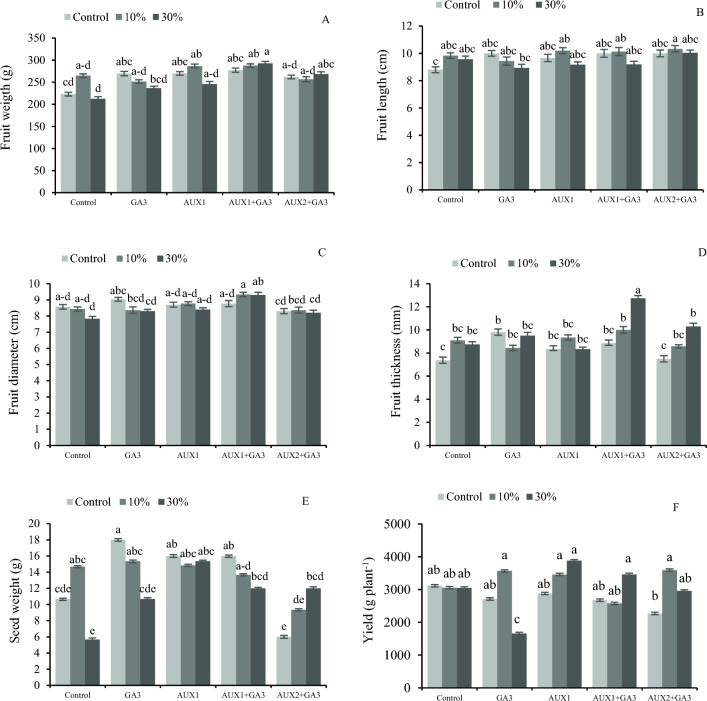
The interaction effect of different phytohormones and pruning levels on fruits characteristics and yield of bell pepper. Treatments: control, GA_3_ (10 µM), AUX1 (10 µM), AUX1 + GA_3_, AUX2 (20 µM) + GA_3_ and pruning treatments: including non-pruning (Control), pruning 10% and 20%. Significant differences are marked by different letters at (*p* < 0.05) according to the least significant difference test (LSD).

The results of the current study indicate that the combinational application of phytohormones and pruning had a significant effect on fruit yield. Specifically, the highest fruit weight was obtained after 30% pruning and the foliar application of AUX1 + GA_3_ treatment compared with the non-pruned and no phytohormone treated control. The lowest fruit weight however, was observed after 30% pruning without phytohormone treatments (Fig. 1A). Fruit length exhibited a general increase in response to the 10% pruning treatment with all phytohormonal treatments. The highest fruit length was recorded after 10% pruning and AUX2 + GA_3_ treatment, while the lowest fruit length was observed after only 30% pruning (Fig. 1B).

The 10% pruning along with AUX1 + GA_3_ resulted in the highest fruit diameter, while 30% pruning without any phytohormonal treatment resulted in the lowest fruit diameter (Fig. 1C). The results also revealed a significantly higher fruit thickness after 30% pruning and AUX1 + GA_3_ treatment, exhibiting a 73% increase compared with the control. Conversely, the lowest fruit thickness was observed in the non-pruned treatments, particularly in the AUX2 + GA_3_ treated plants (Fig. 1D). In general, we can state that a highest seed weight was observed in most hormonal treatments independent of pruning. Particularly, the GA_3_, AUX1 + GA_3_, and Aux1 treatments exhibited a highest seed weight, while the lowest seed weight was measured in the 30% pruning treatment without phytohormones application (Fig. 1E). It was observed that both pruning levels increased yield compared with the control, although this increase was not statistically significant. The lowest yield was obtained from plants under 30% pruning and with only GA_3_ application (Fig. 1F).

### Interacting effects of pruning and phytohormones on the firmness and color traits of bell pepper fruits

**Figure 2:**
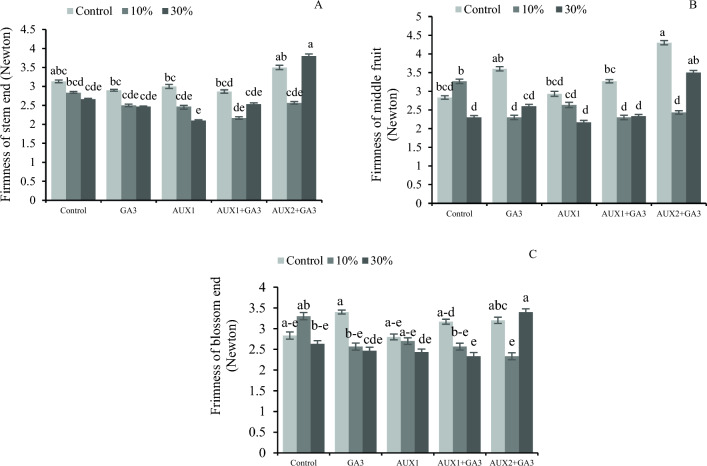
The interaction effect of different phytohormones and pruning levels on firmness of bell pepper. Treatments: control, GA_3_ (10 µM), AUX1 (10 µM), AUX1 + GA_3_, AUX2 (20 µM) + GA_3_ and pruning treatments: including non-pruning (Control), pruning 10% and 20%. Significant differences are shown by different letters at (*p* < 0.05) according to the least significant difference test (LSD).

Pruning and phytohormone treatments interacted to affect fruit firmness, as shown by the findings of this study. More specifically, both 30% pruning plus AUX2 + GA_3_ and AUX2 + GA_3_ without pruning resulted in the firmest plants. On the other hand, Fig. 2A shows that the firmness of the stem end was decreased in pruning 30% in AUX1.The results indicate that the firmness of the fruit middle region was significantly influenced (*P* < 0.05) by hormonal treatments, particularly with the application of AUX 2 + GA_3_ and no pruning. Conversely, the lowest level of firmness of the middle fruit was observed in the case of 30% pruning with AUX1 and AUX1 + GA_3_ (Fig. 2B). The results indicate that the highest level of firmness blossom end of the fruit was observed in the treatments without pruning, while the lowest level was observed in the 30% pruning with AUX1 + GA_3_ treatment (Fig. 2C).

**Figure 3:**
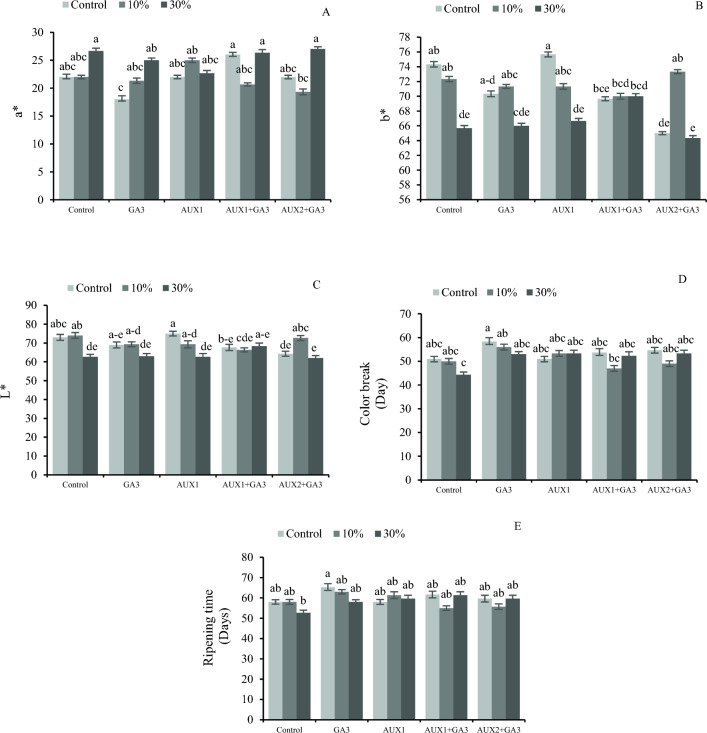
The interaction effect of different phytohormones and pruning levels on ripening time and color index of bell pepper. Treatments: control, GA_3_ (10 µM), AUX1 (10 µM), AUX1 + GA_3_, AUX2 (20 µM) + GA_3_ and pruning treatments: including non-pruning (Control), pruning 10% and 20%. Significant differences are shown by different letters at (*p* < 0.05) according to the least significant difference test (LSD).

The results indicate an interaction effect between pruning and phytohormone treatments on the color index of the plants. In particular, the highest a* color index was observed 30% pruning along with the application of AUX1 + GA_3_ and AUX2 + GA_3_ (Fig. 3A). However, pruning at 30%, along with the application of AUX2 + GA_3,_ showed the lowest values for the b* and L* indices (Fig. 3B, C).

The study on the interplay between pruning and phytohormone impact on the duration of fruit coloration and ripening revealed that no-pruning and GA_3_ application resulted in the longest time for both coloration and ripening (Fig. 3D, E).

### Interaction of pruning and phytohormones on RWC and the concentration of photosynthetic pigment of the bell peppers

**Figure 4:**
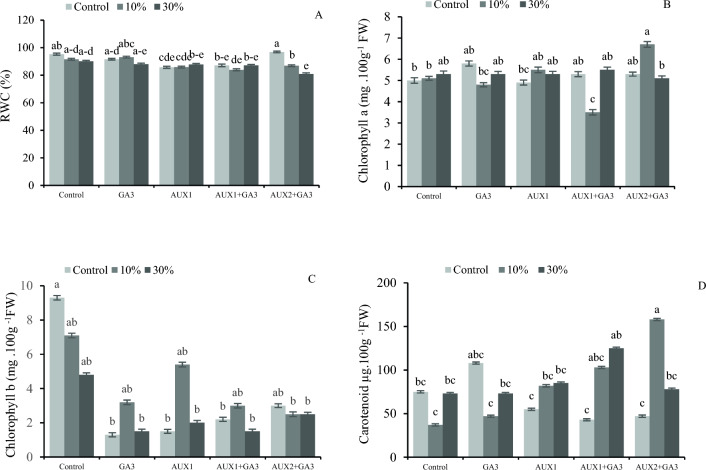
The interaction effect of different phytohormones and pruning levels on RWC and photosynthetic pigments of bell pepper. Treatments: control, GA_3_ (10 µM), AUX1 (10 µM), AUX1 + GA_3_, AUX2 (20 µM) + GA_3_ and pruning treatments: including non-pruning (Control), pruning 10% and 20%. Significant differences are shown by different letters at (*p* < 0.05) according to the least significant difference test (LSD).

The mutual effect of pruning and phytohormones increased the relative water content in non-pruned and AUX2 + GA_3_-treated plants (Fig. 4A). The results indicate that the pruning treatment at 10% plus AUX2 + GA_3_ application exhibited the highest level of chlorophyll *a*, whereas the lowest level was observed in the plants after 10% pruning plus AUX1 + GA_3_ application (Fig. 4B). In addition, the control plants had the highest amount of chlorophyll *b*. The application of pruning and foliar phytohormone treatments resulted in a reduction in chlorophyll *b* levels (Fig. 4C). The results indicate that the highest concentration of carotenoid was observed at a 10% pruning level in addition to AUX2 + GA_3_ treatment, which was significantly higher than the controls (Fig. 4D). Statistical analysis of the data indicates that the combined influence of pruning and phytohormones does not exhibit a significant interaction effect on the total carbohydrate content (Table 2).

### Effects of pruning and phytohormones on the concentrations of vitamin C, TSS, and TA and the antioxidant capacity of bell pepper fruits

**Figure 5:**
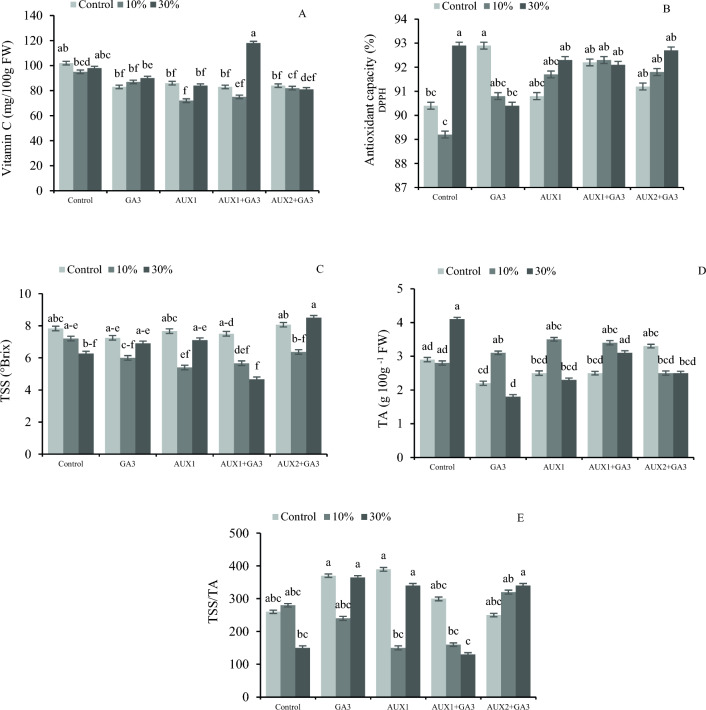
The interaction effect of different phytohormones and pruning levels on vitamin C, antioxidant capacity, total soluble solids (TSS), organic acids (TA), and fruit flavor (TSS/TA) of bell pepper. Treatments: control, GA_3_ (10 µM), AUX1 (10 µM), AUX1 + GA_3_, AUX2 (20 µM) + GA_3_ and pruning treatments: including non-pruning (Control), pruning 10% and 20%. Significant differences are shown by different letters at (*p* < 0.05) according to the least significant difference test (LSD).

The results indicate that the application of 30% pruning plus the AUX1 + GA_3_ treatment resulted in the highest vitamin C content, exhibiting a 63% increment compared with to 10% pruning × AUX1 (Fig. 5A). The results indicate that the application of 30% pruning with no phytohormone treatment, and the non-pruned but GA_3_-supplemneted plants exhibited the highest antioxidant capacity. Conversely, the lowest antioxidant capacity was observed in 10% pruning without phytohormone treatments (Fig. 5B. The content of soluble solids in non-pruned plants treated with different phytohormones, did not exhibit significant differences. However, 30% pruning along with AUX1 + GA_3_ demonstrated the lowest amount of soluble solids (Fig. 5C). The results indicate that the 10% pruning treatment exhibits the highest concentration of organic acid, whereas the GA_3_ treatment and the 30% pruning display the lowest concentration of organic acids (Fig. 5D). Data analysis illustrating the interplay between pruning and phytohormones revealed that the most superior fruit flavor was observed in the GA_3_ and AUX1 treatments, as well as the GA_3_, AUX1, and AUX2 + GA_3_ treatments and the 30% pruning (Fig. 5E).

### The PCA analysis of the investigated traits

**Figure 6:**
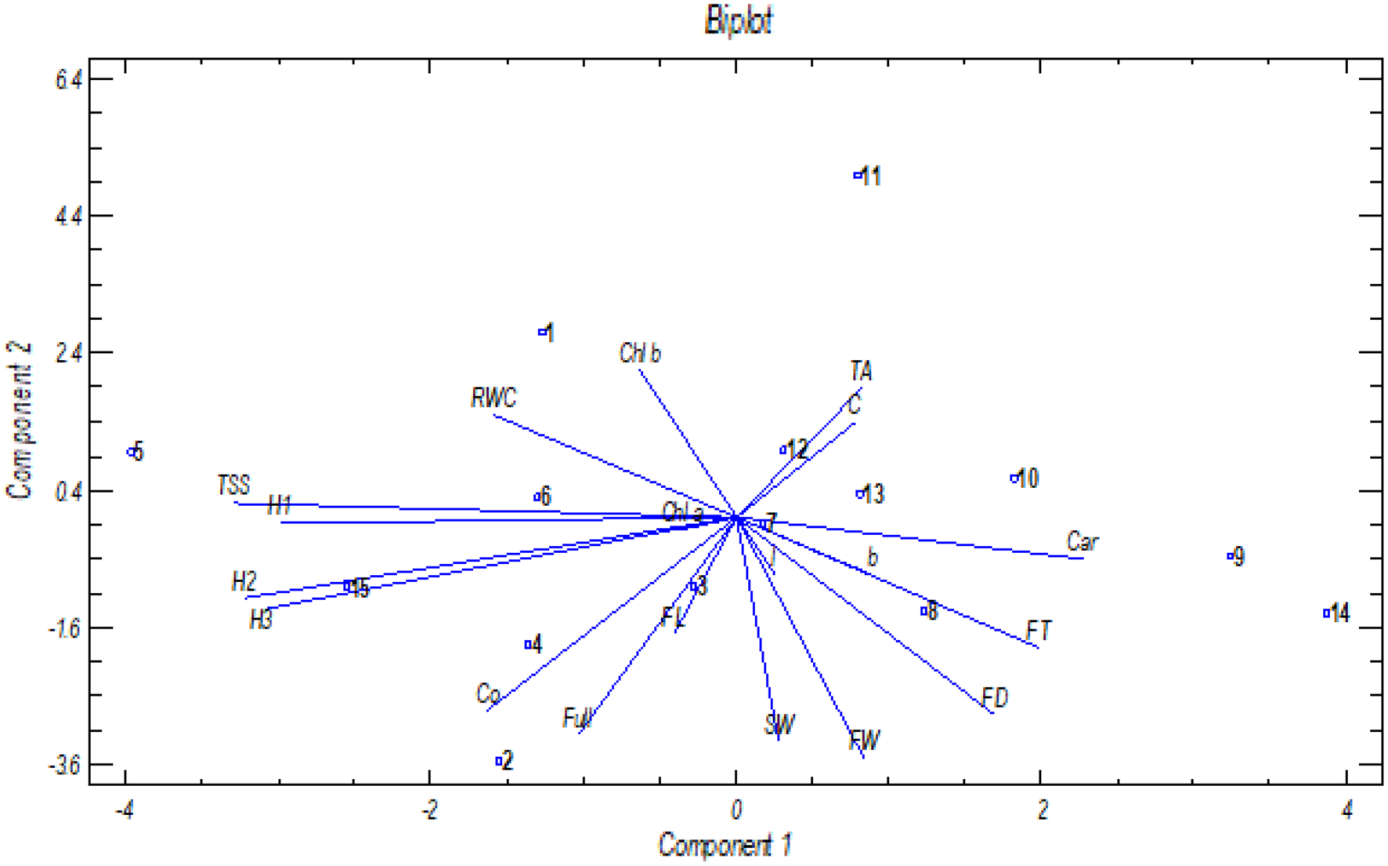
Principal component analysis (PCA) biplot for pruning and phytohormones interaction variables of bell peppers. Vector captions: Fruit weight (FW), fruit length (FL), fruit diameter (FD), TSS (TSS), firmness of stem end (H1), firmness of fruit (H2), firmness blossom end (H3), RWC (RWC), seed weight (SW), b* (b), L* (l), color break (Co), Ripening time (Full), Chlorophyll *a* (Chl *a*), Chlorophyll *b* (Chl *b*), Carotenoid (Car), Vitamin c (C), Total acid (TA). Control × Control (1), control × GA_3_ (2), control × AUX1 (3), control × AUX1 + GA_3_ (4), control × AUX2 + GA_3_ (5), 10% pruning × Control (6), 10% pruning × GA_3_ (7), 10% pruning × AUX1 (8), 10% pruning × AUX1 + GA_3_ (9), 10% pruning × AUX2 + GA_3_ (10), 30% pruning × Control (11), 30% pruning × GA_3_ (12), 30% pruning × AUX1 (13), 30% pruning × AUX1 + GA_3_ (14), 30% pruning × AUX2 + GA_3_ (15).

To summarize the main points of the complex relationship of the examined traits under pruning and phytohormone treatments, a principal component analysis was performed to visualize the results in a bi-plot (Fig. 6) with two principal components (PC1 and PC2). The blue lines in the plot represent the measured variables and the magnitude of the lines indicate the strength of their contribution to each PC. Lines with a tendency toward similar or opposite directions indicates positively or negatively correlated traits. Based on the bi-plot analysis, it appears that the implementation of AUX2 + GA_3_ in the 10% pruning of fruit resulted in a decreased angle in relation to most fruit quality parameters when compared with the control group. When considering the individual effects of GA_3_, AUX1, and AUX2, it can be observed that they are located in the right part of the figure, quite distant from most fruit quality parameters (Fig. 6).

### The heat map graph of the investigated traits

**Figure 7:**
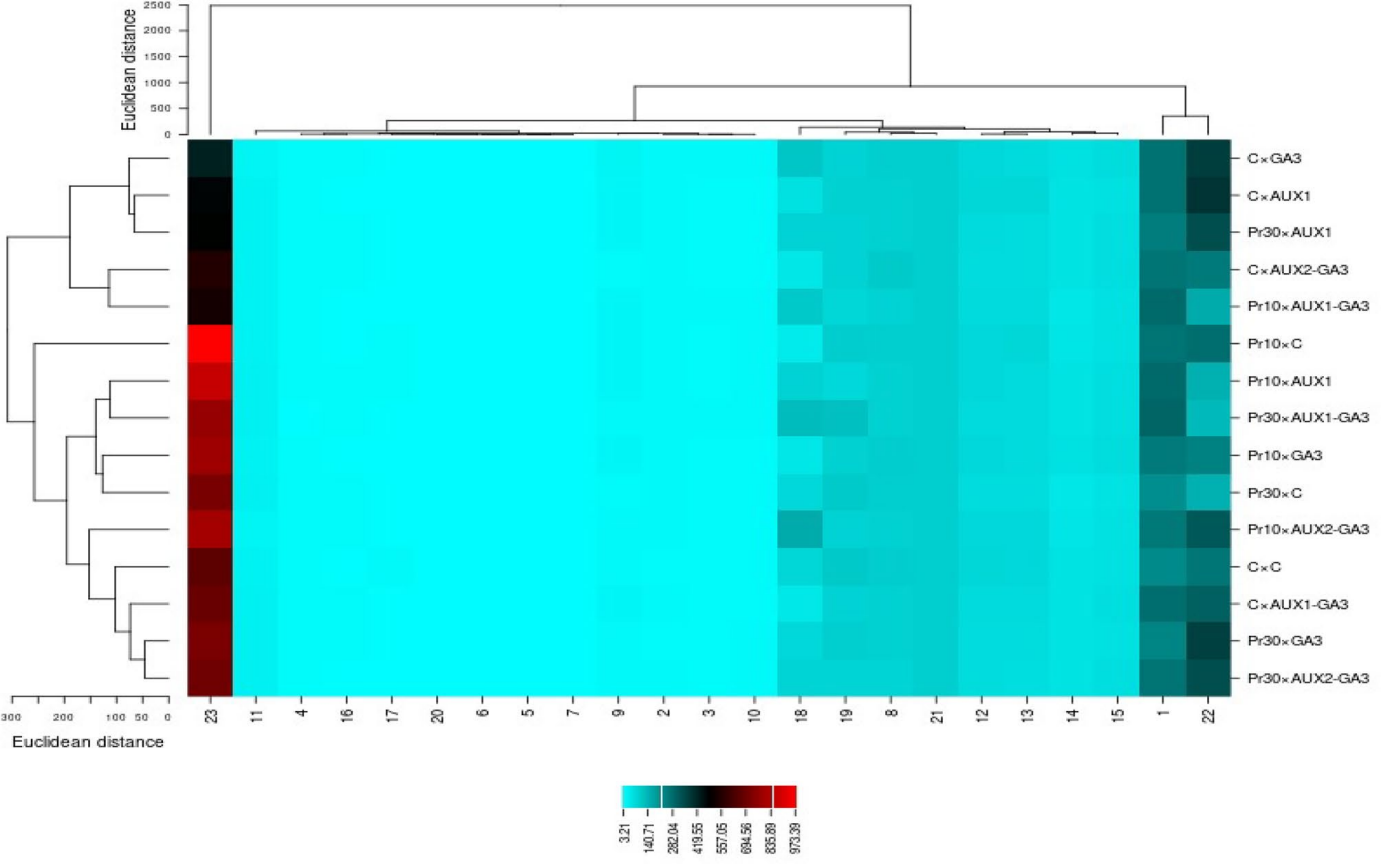
The heat map graphs of interaction of different phytohormones and pruning levels on some characteristics of bell pepper. Fruit weight (1), fruit length (2), fruit diameter (3), TSS (4), firmness of stem end (5), firmness of fruit (6), firmness blossom end (7), RWC (8), seed weight (9), fruit thickness (10), a* (11), b* (12), L* (13), color break (14), ripening time (15), chlorophyll a (16), chlorophyll b (17), carotenoid (18), vitamin C (19), total acid (20), antioxidant capacity (21), fruit flavor (22), yield (23). Control × Control (C × C), control × GA_3_ (C × GA_3_), control × AUX1 (C × AUX1), control × AUX1 + GA_3_ (C × AUX1-GA_3_), control × AUX2 + GA_3_ (C × AUX2-GA_3_), 10% pruning × Control (Pr10 × C), 10% pruning × GA_3_ (Pr10 × GA3), 10% pruning × AUX1 (Pr10 × AUX1), 10% pruning × AUX1 + GA_3_ (Pr10 × AUX1-GA_3_), 10% pruning × AUX2 + GA_3_ (Pr10 × AUX2-GA_3_), 30% pruning × Control (Pr30 × C), 30% pruning × GA_3_ (Pr30 × GA_3_), 30% pruning × AUX1 (Pr30 × AUX1), 30% pruning × AUX1 + GA_3_ (Pr30 × AUX1-GA_3_), 30% pruning × AUX2 + GA_3_ (Pr30 × AUX2-GA_3_).

According to the results of the Heat map graph, fruit flavor, fresh weight, carotenoids and vitamin C were affected more by treatments. In general, yield as an important index was isolated in a cluster and the highest rate was observed with pruning application of 10%, so that yield increased in 10% pruning × C, 10% pruning × AUX1, 10% pruning × GA_3_ and 10%pruning × AUX2 + GA_3_ treatments. 30% pruning × AUX1 + GA_3_ treatment increased fresh fruit weight and carotenoid and vitamin C levels. Among the studied traits, fruit firmness and chlorophyll were less affected by treatments. (Fig. 7).

## Discussion

### Effect of pruning and hormone on some morphological characteristics of bell pepper

The evaluation of sweet pepper fruit quality by consumers is based on various factors such as weight, pericarp thickness, color, and nutritional value. Typically, consumers tend to favor fruits that are heavier, thicker, exhibit full ripeness with vibrant coloration, and preferably have higher nutritional values^38–40^. Formerly, the application of natural biostimulant^41^, foliar fertilization^42^, and exogenous plant growth regulators^43^ have been implemented as methods to enhance the qualitative traits of vegetable products.

The visual characteristics of bell peppers, comparable to other horticultural products, are a crucial aspect of their marketing. This encompasses the fruit's size, shape, and uniformity^44^. Tiwari et al.^4^ discovered that the application of auxin results in a significant increase in fruit weight. Furthermore, the concurrent use of both Auxin and GA_3_ also leads to a substantial increase in fruit weight, which agrees with the findings of our study (Fig. 1), where the maximum fruit length and fruit weight were obtained after 10% pruning. The lower fruit weight that has been observed after 30% pruning is because more severe pruning such as 30%, and the subsequent reduction of fruits per plant may contribute to a faster ripening time, or entering the reproductive phase that can impede the vegetative growth of the remaining fruits on the plant^45^. The ideal objective of pruning is to achieve a suitable equilibrium between the quantity and size of fruit by retaining a suitable quantity of branches on the plant. The applied pruning system in our experiment resulted in the attainment of the greatest fruit diameter. As the degree of pruning escalated in plants pruned into two, three, or four branches, there was an observable increase in fruit size, fruit diameter, and average fruit weight. The hypothesized reason for this phenomenon may be the higher leaf-to-fruit ratio in plants subjected to more extensive pruning^46^.

The application of 30% pruning resulted in the minimum quantity of seeds (Fig. 1E). The applied pruning also resulted in the highest thickness of the fruit flesh, which agrees with previous research indicating the linear relationship between the lower fruit and seed number (because of pruning) and the bigger and fleshier fruits^48^. The lower seed yield is also thought to be linked to a greater allocation of nutrients toward the fruit flesh, leading to an increased fruit thickness^47^. We found that the application of the AUX2 + GA_3_ phytohormone resulted in the lowest seed weight. This outcome can be attributed to a known hormone-induced parthenocarpy of certain fruits, such as pepper, tomato, and eggplant, which subsequently reduced the number of seeds produced. Parniani et al.^48^ found the highest seed weight of pepper in plants without pruning and the lowest seed weight was observed when five and seven fruits were kept per plant. The results of our research also showed that 30% pruning decreased the seed weight compared to the no pruning treatment. The study conducted by Flores-Velazquez et al.^49^ revealed that treatments featuring three stems per plant exhibited superior yields in comparison to treatments featuring two stems but no notable distinctions were observed among the various interventions. According to the study conducted by Sanchez-del Castillo et al.,^50^ an increase in plant density resulted in a decrease in both fruit yield and quality. Their findings indicated a positive correlation between the number of stems per plant and the number of fruits produced, while concurrently revealing a negative correlation between fruit size and stem quantity.

In our study, the firmness of pepper tissues (blossom end, mid-stem, and stem), was higher in the control plants. Severe pruning, influenced the firmness of the fruit, which is probably due to accelerated ripening and dehydration. Pruning resulted in a substantial reduction in the relative water content of the plants. According to Park and Malka^51^, the exogenous application of high concentration gibberellin before harvesting resulted in a longer green color maintenance and a delay in fruit ripening by six days. In our experiment, a significantly earlier color break was observed only because of 30% pruning and no significant differences were detected in color break time due to the application GA_3_ (Fig. 3). However, the coloration pattern and pigment distribution in the pepper fruits were significantly influenced by different pruning systems and planting distance^46^.

### Effect of pruning and hormone on some physiological characteristics of the bell pepper

The findings of this study indicate that the pruning had a significant impact on chlorophyll a and chlorophyll b levels, as manifested by their consistent high levels across all pruning levels. It was also found that the application of AUX1 × GA_3_ resulted in a significant reduction in chlorophyll levels, when compared to control plants. Considering the interactive effects pruning and phytohormones application on pigments, the results suggest that the highest level of chlorophyll a was associated with a pruning level of 10% and AUX2 + GA_3_ application. The elevated light exposure consequent to pruning, specifically at 30%, appeared to have influence on chlorophylls content and composition. The treatments without pruning exhibited a greater concentration of chlorophyll, which may be attributed to a heightened requirement for assimilates and, consequently, an increase in chlorophyll production and photosynthesis. The findings of this study indicate a significant interaction between the applied pruning and phytohormones, wherein the application of 10% pruning and AUX2 + GA_3_ during pruning resulted in the highest concentration of carotenoids. The implementation of optimal pruning techniques, and the subsequent increase in light exposure, has been observed to enhance the photosynthetic activity and improve nutrient availability for optimum fruit development. Additionally, the application of phytohormones has been found to promote carotenoid accumulation and facilitate fruit ripening^52^. The use of 10% pruning together with the simultaneous application of both AUX and GA_3_ has yielded favorable results. According to Chen et al.^53^, supplementation of phytohormones can stimulate the accumulation of carotenoids. Nevertheless, the extent of carotenoid augmentation is contingent upon the particular phytohormonal intervention.

Previous studies have reported that the application of gibberellic acid enhanced the growth and performance of chili peppers. This effect is attributed to a more efficient utilization of assimilates by plants, leading to an increased photosynthetic efficiency, resource availability, translocation, and ultimately, higher concentrations of sugars, capsaicinoids, and carotenoids^54^. According to our findings, the pruning was found to yield the highest concentration of vitamin C only when it was combined with the effect of supplemented AUX1 + GA_3_. This is perhaps attributed to the significant impact of light intensity on the biosynthesis of vitamin C. Singh and Kaur^55^ found that plants pruned to four branches exhibit lower levels of vitamin C. We also found a slight decrease (but not significant) in vitamin C content as a results of pruning. We can state that the application of gibberellin at an appropriate developmental stage of sweet pepper plants not only enhances the quality of the product but also contributes to the enhancement of vitamin C levels in the fruit. The elevation observed in the quantity of ascorbic acid subsequent to the application of gibberellic acid is likely attributable to an increase in the biosynthesis of ascorbic acid and its metabolism. The research on investigation the impact of pre-harvest and post-harvest variables on the quantity of vitamin C in horticultural products is suggesting a number of factors, including genotype, pre-harvest climatic conditions, cultivation techniques, harvesting procedures, and storage condition, to be influential in this regard. Post-harvest handling practices can affect the concentration of vitamin C present in fruits and vegetables. Additionally, thinning and pruning may have an impact on the quantity of this vitamin as well^56^.

Carbohydrates are derived through the reduction of carbon dioxide during the process of photosynthesis. The impact of pruning and phytohormone application on the overall carbohydrate level of bell pepper plants was also investigated in this study. The results indicate that while AUX2 + GA_3_ phytohormone exhibited slightly higher carbohydrates content, where neither the pruning nor the hormone treatment had a significant effect on the carbohydrate level of the plants (data are not shown). Although the allocation of assimilates (carbohydrate content, sugar, and starch) is subject to competition between vegetative and reproductive organs, many of findings indicate that the leaves exhibit a higher accumulation of sugars compared to the reproductive parts.

The utilization of NAA results in an elevation of acidity and TSS levels in tomatoes^57^. The application of NAA in tomatoes also resulted in a significant increase in total soluble solids content, as reported by Gupta et al.^58^) and Pudir and Yadav^59^. The fruit obtained from the plant subjected to 50 ppm NAA exhibited the greatest concentration of vitamin C. The positive impact of 100 ppm NAA on the total soluble solids (TSS) of the fruit was also noted. The application of NAA at different concentrations was found to have a significant impact on the nutrient, chlorophyll, and sugar contents of tomato fruits^60^. In our study, the foliar application of AUX1 + GA_3_ also increased the carotenoid and TSS. According to Parniani et al.^48^, the translocation of assimilates and sugar (TSS) to the fruit was found to be better in fruit-pruned plants even under low light conditions. The present investigation revealed that less intensive pruning resulted in elevated levels of soluble solids in comparison to 30% pruning which is in contrast with the findings of Barzegar et al.^61^, where the change in plant density did not have a significant impact on the soluble solids of pepper fruit. When melon plants were subjected to varying concentrations of AUX (25, 50, and 100 mg L^-1^), the increase in soluble solids positively correlated with the increase in AUX concentration^61^. We also observed a substantial enhancement in dissolved solids content of bell pepper fruits after the application of AUX2 in conjunction with gibberellin (Fig. 5).

Considering the interplay effect of pruning and hormone in relation to organic acid levels revealed that the highest concentration of organic acid was determined in plants subjected to 30% pruning. It is already recognized that the absence of plant hormones results in an elevation of organic acid levels. Phytohormones play a crucial role in the metabolism of sugars and the ripening of fruits, whereby organic acids are transformed into sugars during the ripening process. Research studies have demonstrated that the application of auxin and gibberellin induces diverse alterations in fruit growth, including morphological, histological, and sugar metabolism, as well as changing the organic acids level^62^. The analysis of the obtained results revealed the highest antioxidant capacity being detected equally after 30% pruning or GA_3_ treatment. According to Talat et al.^52^, the application of gibberellic acid at a concentration of 25 and 45 ppm, also resulted in an increase in ascorbic acid, antioxidants, phenols, flavonoids and carotenoids content in the mandarin fruits.

## Conclusions

In conclusion, the application of fruit pruning and phytohormone spraying utilizing auxin and gibberellin caused alterations in the majority of the traits analyzed in this study. The results obtained from the study indicate that the application of 10% pruning had a significant impact on the weight of the seeds and the length of the fruit. On the other hand, the implementation of 30% pruning resulted in an increase in the thickness of the fruit and the vitamin C content. The hormone levels of GA_3_ and AUX1 were found to have significant effects on the ripening process of fruits. Specifically, GA_3_ was observed to prolong the duration of coloration and ripening, while Aux1 was found to enhance the color index and increase the seeds weight. The application of AUX2 + GA_3_ resulted in enhancements in the physical attributes of the fruits, including increased length and firmness, as well as improvements in the levels of photosynthetic pigments and soluble solids, fruit length, chlorophyll and carotenoid content. We could show the dynamic reactions of bell pepper fruits to the applied physical and phytochemical treatments and identify the affected traits with potential applicability to enhance the production system according to the marketing requirements and consumer’s expectations.

## Data Availability

All data generated or analyzed during this study are included in this published article. Additional information will be available on request to the corresponding authors.

## References

[1] Marin A, Ferreres F, Tomas-Barberan FA, Gil MI (2004). Characterization and quantitation of antioxidant constituents of sweet pepper (*Capsicum annuum* L.). J. Agric. Food. Chem..

[2] Aminifard M, Bayat H (2016). Effect of vermicompost on fruit yield and quality of bell pepper. Int. J. Hortic. Sci. Technol..

[3] Leyser O (2006). Dynamic integration of auxin transport and signaling. Curr. Biol..

[4] Tiwari A, Offringa R, Heuvelinv E (2012). Auxin-induced fruit set in *Capsicum annuum* L. requires downstream gibberellin biosynthesis. J. Plant. Growth. Regul..

[5] Arteca RN (1996). Plant Growth Substances.

[6] Li J, Guan Y, Yuan L, Hou J, Wang C, Liu F, Zhu S (2019). Effects of exogenous IAA in regulating photosynthetic capacity, carbohydrate metabolism and yield of *Zizania latifolia*. Sci. Hortic..

[7] Singh S, Prasad SM (2015). IAA alleviates Cd toxicity on growth, photosynthesis and oxidative damages in eggplant seedlings. Plant Growth Regul..

[8] Hayat Q, Hayat S, Ali B, Ahmad A (2009). Auxin analogues and nitrogen metabolism, photosynthesis, and yield of chickpea. J. Plant Nutr..

[9] McAdam SAM, Eléouët MP, Best M, Brodribb TJ, Murphy MC, Cook SD, Dalmais M, Dimitriou T, Gélinas-Marion A, Gill WM, Hegarty M, Hofer JMI, Maconochie M, McAdam EL, McGuiness P, Nichols DS, Ross JJ, Sussmilch FC, Urquhart S (2017). Linking auxin with photosynthetic rate via leaf venation. Plant Physiol..

[10] Hayat S, Fariduddin Q, Ali B, Ahmad A (2006). Effect of chloroindoleauxins on the growth and nitrate reductase activity in *Solanum melongena*. Asian J. Plant Sci..

[11] Lopez ML, Peralta-Videa JR, Benitez T, Duarte-Gardea M, Gardea Torresdey JL (2007). Effects of lead, EDTA, and IAA on nutrient uptake by alfalfa plants. J. Plant Nutr..

[12] San-Francisco S, Houdusse F, Zamarreño AM, Garnica M, Casanova E, García-Mina JM (2005). Effects of IAA and IAA precursors on the development, mineral nutrition, IAA content and free polyamine content of pepper plants cultivated in hydroponic conditions. Sci. Hortic..

[13] Pasternak TP, Potters G, Caubergs R, Jansen MAK (2005). Complementary interactions between oxidative stress and auxins control plant growth responses at plant, organ, and cellular level. J. Exp. Bot..

[14] Piotrowska-Niczyporuk A, Bajguz A (2014). The effect of natural and synthetic auxins on the growth, metabolite content and antioxidant response of green alga *Chlorella vulgaris* (Trebouxiophyceae). Plant Growth. Regul..

[15] Iqbal, N., Nazar, R., Khan, M.I.R., Masood, A. & Khan, N.A. Role of gibberellins in regulation of source-sink relations under optimal and limiting environmental conditions. *Curr. Sci*. **100**(7), 998–1007 http://www.jstor.org/stable/24076517 (2011).

[16] Gelmesa D, Abebie B, Desalegn L (2010). Effects of gibberellic acid and 2,4-dichlorophenoxyacetic acid spray on fruit yield and quality of tomato (*Lycopersicon esculentum* Mill.). J. Plant. Breed. Crop. Sci..

[17] Shukla HS, Kumar V, Tripathi V (2011). Effect of gibberellic acid and boron on development and quality of aonla fruits 'Banarasi'. Acta Hortic..

[18] Singh S, Singh T (2021). Effect of gibberellic acid on growth, yield and quality parameters of chilli (*Capsicum annum* L). J. Pharmacogn. Phytochem..

[19] Fenn MA, Giovannoni JJ (2021). Phytohormones in fruit development and maturation. Plant.

[20] Saha P (2009). Effect of NAA and GA3 on yield and quality of tomato (*Lycopersicon esculentum*, Mill.). J. Ecol. Environ..

[21] Netam JL, Sharma R (2014). Efficacy of plant growth regulators on growth characters and yield attributes in brinjal (*Solanum melongena* L.) cv. Brinjal 3112. IOSR-JAVS.

[22] Thakur O, Kumar V, Singh J (2018). A review on advances in pruning to vegetable crops. Int. J. Curr. Microbiol. Appl. Sci..

[23] Alsadon A, Wahb-Allah M, Abdel-Rezzak H, Ibrahim A (2013). Effect of pruning systems on growth, fruit yield and quality traits of three greenhouse-grown bell pepper (*Capsicum annuum* L.) cultivars. Aust. J. Crop. Sci..

[24] Jovicich E, Cantliffe DJ, Stofella PJ (2004). Fruit yield quality of greenhouse-grown bell pepper as influenced by density, container and trellis system. Hortic. Technol..

[25] Rubio JS, García-Sánchez F, Flores P, Navarro JM, Martínez V (2010). Yield and fruit quality of sweet pepper in response to fertilisation with Ca^2+^ and K^+^. Span. J. Agric. Res..

[26] Pathaveerat S, Jantra C, Slaughter DC, Roach A (2018). Development of a hand held precision penetrometer system for fruit firmness measurement. Postharvest. Biol. Technol..

[27] Tian YW, Wang XJ (2009). Analysis of leaf parameters measurement of cucumber based on image processing. WRI World Congr. Softw. Eng..

[28] Lopez-Serrano L, Canet-Sanchis G, Vuletin Selak G, Penella C, San Bautista A, Lopez-Galarza S, Calatayud A (2019). Pepper rootstock and scion physiological responses under drought stress. Front. Plant Sci..

[29] Alpuerto J, Hussain RMF, Fukao T (2016). The key regulator of submergence tolerance, SUB1A, promotes photosynthetic and metabolic recovery from submergence damage in rice leaves. Plant. Cell. Environ..

[30] McCready RM, Guggolz J, Silviera V, Owens HS (1950). Determination of starch and amylose in vegetables. Anal. Chem..

[31] Lichtenthaler HK (1987). Chlorophylls and carotenoids: pigments of photosynthetic biomembranes. Methods. Enzymol..

[32] Njoku NE, Ubbaonu CN, Alagbaoso SO, Eluchie CN, Umelo MC (2015). Amino acid profile and oxidizable vitamin content of *Synsepalum dulcificum* berry (miracle fruit) pulp. Food. Sci. Nutr..

[33] Idris OA, Wintola OA, Afolayan AJ (2019). Comparison of the proximate composition, vitamins (ascorbic acid, α-tocopherol and retinol), anti-nutrients (phytate and oxalate) and the GC–MS analysis of the essential oil of the root and leaf of *Rumex crispus* L. Plants..

[34] Koleva II, Van Beek TA, Linssen JPH, de Groot A, Evstatieva LN (2002). Screening of plant extracts for antioxidant activity: A comparative study on three testing methods. Phytochem. Anal..

[35] El-Mogy MM, Salama AM, Mohamed HFY, Abdelgawad KF, Abdeldaym EA (2019). Responding of long green pepper plants to different sources of foliar potassium fertilizer. Agriculture (Pol'nohospodárstvo).

[36] Ye Z, Shang Z, Zhang S, Li M, Zhang X, Ren H, Hu X, Yi J (2022). Dynamic analysis of flavor properties and microbial communities in Chinese pickled chili pepper (*Capsicum frutescens* L.): A typical industrial-scale natural fermentation process. Food. Res. Int..

[37] Abbas Marhoon I, Kadheem Abbas M (2015). Effect of foliar application of seaweed extract and amino acids on some vegetative and anatomical characters of two sweet pepper (*Capsicum annuum* L.) cultivars. IJRSAS..

[38] Jamiolkowska A, Buczkowska H, Thanoon AH (2016). Effect of biological preparations on content of saccharides in sweet pepper fruits. Acta Sci. Pol. Hortorum Cultus.

[39] Buczkowska, H., Salata, A. & Rozek, E. Diversity of the utility and biological value of fruits of some sweet pepper cultivars. *Acta Sci. Pol. Hortorum Cultus* **13**, 49–62. http://www.acta.media.pl/pl/main.php?p=8&sub=0&act=10&s=7&no=458&lang=pl (2014).

[40] Jadczak D, Grzeszczuk M, Kosecka M (2010). Quality characteristics and content of mineral compounds in fruit of some cultivars of sweet pepper (*Capsicum annuum* L.). J. Elem..

[41] Parađiković N, Vinković T, Vinković Vrček I, Žuntar I, Bojić M, Medić-Šarić M (2011). Effect of natural biostimulants on yield and nutritional quality: An example of sweet yellow pepper (*Capsicum annuum* L.) plants. J. Sci. Food. Agric..

[42] Haytova, D. A review of foliar fertilization of some vegetables crops. *Annu. Res. Rev. Biol*. **3**(4), 455–465 https://journalarrb.com/index.php/ARRB/article/view/24752 (2013).

[43] Pérez-Jiménez M, Pazos-Navarro M, López-Marin J, Gálvez A, Varó P, Del Amor F (2015). Foliar application of plant growth regulators changes the nutrient composition of sweet pepper (*Capsicum annuum* L.). Sci. Hortic..

[44] Navarro JM, Garrido C, Carvajal M, Martinez V (2002). Yield and fruit quality of pepper plants under sulphate and chloride salinity. J. Hortic. Sci. Biotechnol..

[45] Marcelis LF, Heuvelink E, Hofman-Eijer LR, Den BJ, Xue LB (2004). Flower and fruit abortion in sweet pepper in relation to source and sink strength. J. Exp. Bot..

[46] Aydın A, Basak H, Cetin AN (2022). Effects of different pruning systems on fruit quality and yield in California wonder peppers (*Capsicum annuum* L.) grown in soilless culture, Manas. J. Agric. Vet..

[47] Marcelis FM, Baan Hofman-Eijer LR (1997). Effects of seed number on competition and dominace among fruits in *Capsicum annuum* L. Ann. Bot..

[48] Parniani F, Haghighi M, Mireei S (2022). The effect of adjusting fruit loading by pruning on the yield and quality of sweet pepper in low light condition. S. Afr. J. Bot..

[49] Flores-Velazquez J, Mendoza-Perez C, Rubiños-Panta JE, Ruelas-Islas JDR (2022). Quality and yield of bell pepper cultivated with two and three stems in a modern agriculture system. Horticulturae..

[50] Sanchez-del Castillo, F., Moreno-Pérez, E.C., Reséndiz-Melgar, R.C., Colinas-León, M.T. & Rodríguez Pérez, J.E. Bell pepper production (*Capsicum annuum* L.) in short cycles. *Agrociencia* **51**(4), 437–446 http://www.scielo.org.mx/pdf/agro/v51n4/1405-3195-agro-51-04-00437-en (2017).

[51] Park MH, Malka SK (2022). Gibberellin delays metabolic shift during tomato ripening by inducing auxin signaling. Front. Plant Sci..

[52] Talat H, Shafqat W, Qureshi MA, Sharif N, Raza MK, ud Din S, Jaskani MJ (2020). Effect of gibberellic acid on fruit quality of Kinnow mandarin. J. Glob. Innov. Agric. Sci..

[53] Chen JH, Wei D, Lim PE (2020). Enhanced coproduction of astaxanthin and lipids by the green microalga chromochloris zofingiensis: Selected phytohormones as positive stimulators. Bioresour. Technol..

[54] Pichardo-González JM, Guevara-Olvera L, Couoh-Uicab YL, González-Cruz L, Bernardino-Nicanor A, Medina HR, González-Chavira MM, Acosta-García G (2018). Effect of gibberellins on the yield of jalapeño pepper (*Capsicum annuum* L.). Rev. Mexicana. Cienc. Agric..

[55] Singh I, Kaur A (2018). Effect of pruning systems on growth and yield traits of greenhouse grown bell pepper (*Capsicum annuum* L. var. grossum). Indian J. Agric. Res..

[56] Lee SK, Kader AA (2000). Preharvest and postharvest factors influencing vitamin C content of horticultural crops. Postharvest Biol. Technol..

[57] Patel JS, Sitapara HH, Patel KA (2012). Influence of plant growth regulators on growth, yield and quality of tomato and brinjal. Int. J. For. Crop. Improv..

[58] Gupta PK, Gupta AK, Reddy S (2003). Response of plant growth regulators and micronutrient mixtures on fruit size, color and yield of tomato (*Lycopersicon esculentum* Mill.). Ann. Agric. Sci..

[59] Pudir JPS, Yadav PK (2001). Note on effect of GA_3_ NAA and 2, 4-D on growth, yield and quality of tomato var Punjab Chuhara. Curr. Agric..

[60] Alam SM, Khan MA (2002). Fruit yield of tomato as affected by NAA spray. Asian J. Plant Sci..

[61] Barzegar, T., Eliyasi Moghaddam, M. & Ghahremani, Z. Effect of foliar application of naphthalene acetic acid and plant thinning on sugar contents of melon (*Cucumis melo*) fruit cv. Khatooni, Iran. *J. Plant Physiol*. **5**(2), 1281–1287. https://ijpp.saveh.iau.ir/article_539655_271b7cd8d067f07d2c7de293206b756b.pdf (2015).

[62] de Jong M, Mariani C, Vriezen WH (2009). The role of auxin and gibberellin in tomato fruit set. J. Exp. Bot..

